# Bullous Lupus: An Atypical Case of Refractory Disease in a Patient with Sulfa Allergy

**DOI:** 10.1155/2020/8873337

**Published:** 2020-07-25

**Authors:** Maria Salgado Guerrero, Oscar Mena Miranda, Ana B. Arevalo, Nevena Barjaktarovic, Barbara Mendez

**Affiliations:** ^1^Department of Medicine, Jacobi Medical Center/Albert Einstein College of Medicine, Bronx, NY, USA; ^2^Department of Medicine, Mount Sinai Morningside-West, New York, NY, USA; ^3^The Wright Center for Community Health, Scranton, PA, USA; ^4^Division of Rheumatology, Jacobi Medical Center/Albert Einstein College of Medicine, Bronx, NY, USA

## Abstract

Bullous systemic lupus erythematosus (BSLE) is a rare cutaneous autoimmune disorder characterized by rapid, widespread vesiculobullous lesions in patients with Systemic Lupus Erythematosus (SLE). BSLE can present as the initial manifestation of SLE and may be a marker of severe disease. In this case report, we present a case of a 22-year-old African American woman with BSLE and impaired renal function with subsequent nephrotic range proteinuria concerning for lupus nephritis and autoimmune hemolytic anemia, refractory to systemic corticosteroids, immunoglobulin, and mycophenolate mofetil, requiring dapsone after careful desensitization due to prior history of angioedema with sulfa drugs. This case highlights the importance of the prompt recognition of BSLE as the initial manifestations of SLE and illustrates the association of BSLE with severe disease and the benefit of concomitant use of dapsone with corticosteroids and other immunosuppressant drugs, even in patients with a history of sulfa allergy.

## 1. Introduction

Bullous systemic lupus erythematosus (BSLE) is a rare autoimmune blistering disorder most commonly seen in patients with preceding systemic lupus erythematosus (SLE); however, it can also present as the initial manifestation of SLE [1–4]. The correlation between BSLE and SLE activity remains controversial. Nonetheless, recent evidence shows that BSLE is rarely isolated and is usually associated with extracutaneous manifestations suggestive of severe disease [5]. Dapsone is considered first-line treatment. In cases of contraindication or systemic SLE manifestations, immunosuppressive drugs and corticosteroids can be used [6–8]. We present a case of BSLE with concomitant lupus nephritis and autoimmune hemolytic anemia refractory to systemic corticosteroids and other immunosuppressant drugs, requiring dapsone after desensitization due to prior history of angioedema with sulfa drugs.

## 2. Case Report

### 2.1. Case Presentation

**Figure 1:**
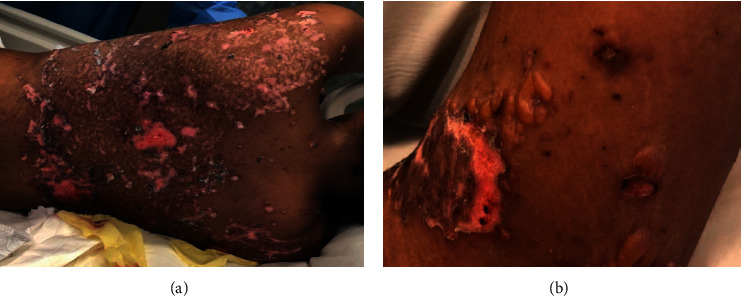
Tense bullae over erythematous plaques on the trunk (a) and left upper extremity (b).

A 22-year-old African American woman presented with a three weeks of a widespread blistering eruption on the trunk, face, and extremities after sun exposure at an outdoor concert. Her medical history included Von Willebrand disease, eczema, and a documented sulfa allergy reaction (angioedema and rash). Family history was negative for autoimmune diseases. On review of systems, the patient denied a history of photosensitivity, polyarthralgia, or oral ulcers, but reported fever and malaise four days prior to presentation. Physical examination was notable for fever (temp 102.8°F/39.3°C) and clear fluid-filled bullae over the face, trunk, and extremities with crusting lesions on the lips, soft palate, and genitals (Figure 1). No lymphadenopathy or synovitis was detected. The remaining physical examination was normal.

**Figure 2:**
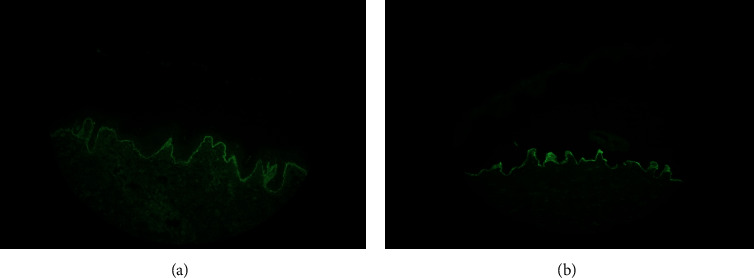
Direct immunofluorescence with linear deposition of IgG and IgA at the basement membrane zone (a) and salt-split preparation with immune complex deposition at the dermal site (b).

Initial laboratory evaluation revealed a hemoglobin level of 9.1 g/L (reference range, 12–16 g/L), leukocytes of 6.4 × 10^9^/L (reference range, 3.5–11 × 10^9^/L), and platelet count of 131 × 10^9^/L (reference range, 150–440 × 10^9^/L). Coombs assay, lactate dehydrogenase, and haptoglobin were at normal levels. A comprehensive metabolic panel revealed a creatinine of 2.7 mg/dL (reference range, 0.1–1.5 mg/dL) and blood urea nitrogen of 45 mg/dL (reference range, 5–20 mg/dL). Urinalysis showed the presence of proteinuria, with no hematuria, leukocyturia, or casts. The spot urine protein/creatinine ratio was 78.7 mg/mmol (reference range, <3 mg/mmol). An echocardiogram showed moderate pericardial effusion with no evidence of hemodynamic compromise. Subsequent laboratory results revealed low complement component 3 and complement component 4 levels, positive antinuclear antibody (ANA) (1 : 1280 speckled pattern, reference range >1 : 40), positive anti-Smith antibody (anti-Sm), anti-Ribonucleoprotein (anti-RNP), anti-Sjogren's Syndrome-related antigen A (anti-SSA), anti-Sjogren's Syndrome-related antigen B (anti-SSB), double-stranded DNA (490 IU/mL) (dsDNA, reference range, <30 UI/mL), and anti-Histone, confirming the diagnosis of SLE by the American College of Rheumatology (ACR) [9, 10] and Systemic Lupus International Collaborating Clinics (SLICC) criteria [11]. Lupus anticoagulant and anti-cardiolipin antibodies were negative as part of the screening for antiphospholipid syndrome. Skin histopathology revealed subepidermal neutrophils infiltrate. Direct immunofluorescence (IF) showed linear deposition of IgG and IgA at the dermal side with salt-split skin preparation consistent with BSLE (Figure 2).

### 2.2. Treatment and Hospital Course

**Figure 3:**
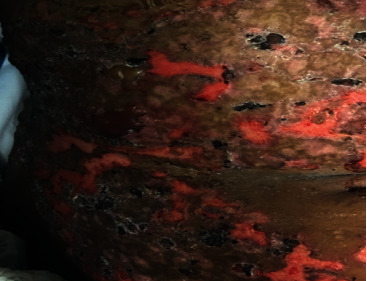
Worsening blistering eruption on the abdomen.

The patient was initially treated with pulse dose of methylprednisolone (1 mg/kg/day) for three days followed by oral prednisone 60 mg daily and intravenous immunoglobulin (2 g/kg) over three days. A repeated spot urine protein/creatinine ratio showed worsening proteinuria (248.6 mg/mmol). Urine microscopy revealed dysmorphic red blood cells with negative Antineutrophil Cytoplasmic Antibodies (ANCA) studies. Renal biopsy was deferred due to the worsening of skin lesions and the patient's inability to prone. Mycophenolate mofetil was started given high suspicion for lupus nephritis. Despite aggressive treatment, new blistering lesions continued to appear on the face (at the vermilion border of the lips and the nasal bridge), trunk, abdomen, and limbs (Figure 3). The decision for starting dapsone was made after confirming normal glucose-6 phosphate dehydrogenase levels and after careful sulfa desensitization using the 12-step protocol for patients with immediate hypersensitivity drug reactions [12]. After successful desensitization, dapsone was started at 12.5 mg, followed by 25 mg the next day, and subsequently increased to 50 mg daily with significant improvement of the skin lesions after one week of therapy. Although skin lesions started to improve, the patient's anemia worsened. There was suspicion for dapsone-related hemolytic anemia; however, further workup revealed positive Coombs testing consistent with warm autoimmune hemolytic anemia requiring rituximab with subsequent stabilization of hemoglobin levels. Mycophenolate mofetil was stopped during rituximab therapy.

### 2.3. Outcome and Follow-Up

**Figure 4:**
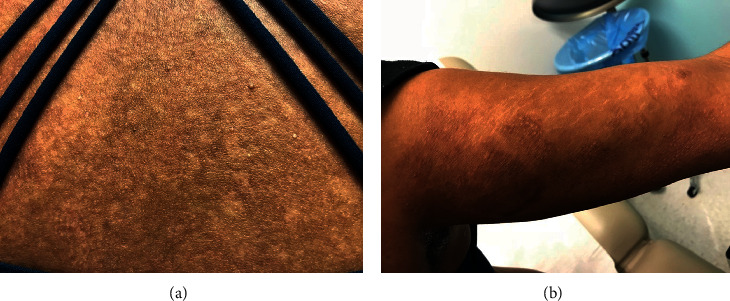
Hypopigmented macules on the trunk (a) and upper extremity (b) with no bullae lesions after seven months of treatment with dapsone.

The patient was discharged home after four weeks of hospital stay. On a six weeks' postdischarge follow-up, dapsone was tapered down from 50 mg daily to 25 mg daily, given the improvement of skin lesions. Mycophenolate mofetil was restarted after rituximab induction dose (dose protocol of 375 mg/m^2^, given over four weeks) with complete remission of renal disease after twenty weeks of discharge. Prednisone was tapered down from 40 mg to 5 mg daily by week twenty-four. The patient was last seen after thirty weeks of discharge. The skin lesions showed a marked improvement with only few hypopigmented macules on the trunk and upper extremities (Figure 4). There were no signs or symptoms of active lupus at that time. There was no evidence of proteinuria or anemia. Prednisone was kept at 5 mg daily with the plan to continue taper in the following weeks.

## 3. Discussion

BSLE is a rare manifestation of SLE that mainly affects African American women in their second to fourth decades of life [2]. The presentation is often rapid and characterized by widespread fluid-filled tense bullae affecting inflamed or normal skin, with a preference for sun-exposed areas including the trunk, upper extremities, neck, face, vermillion border, and oral mucosa. Lesions mostly heal without leaving a scar; however, hypopigmented lesions can appear over recovered skin [1, 3, 4].

The diagnostic criteria of BSLE were first described by Camisa and Sharma in 1983 [13] and later revised in 1988 after the use of IF [14]. They include (1) diagnosis of SLE based on American College of Rheumatology (ACR) criteria; (2) vesicles and/or bullae; (3) histopathologic features similar to dermatitis herpetiformis; (4) direct IF with IgG and/or IgM and often IgA at the basement membrane zone; and (5) indirect IF testing that can be negative or positive for circulating autoantibodies against the basement membrane zone via the salt-split skin technique. Our patient fulfilled both the ACR criteria for SLE and BSLE criteria that were later confirmed with the skin biopsy results.

Due to the similar appearance of skin lesions, BSLE can be easily confused with other subepidermal bullous diseases such as bullous pemphigoid (BP), epidermolysis bullosa acquisita (EBA), linear IgA bullous dermatosis, and dermatitis herpetiformis (DH) [1, 3, 4]. Other less common skin lesions similar to BSLE include pemphigus vulgaris (PV), pemphigus foliaceus (PF), pemphigus erythematosus (PE), pemphigus herpetiformis (PH), paraneoplastic pemphigus (PNP), and mucous-membrane pemphigoid (MMP) [15]. Therefore, both histology and IF are essential in the diagnosis of these entities. The histopathology of BSLE usually shows subepidermal bullae with neutrophilic infiltrate, dermal edema, and large deposits of mucin. The presence of microabscesses in the dermal papillae is also frequently seen, which makes BSLE very similar to DH. However, the neutrophils tend to be in a dispersed arrangement and may extend into the papillary dermal collagen in BSLE, whereas in DH, the neutrophilic infiltrate is focal and localized at the papillary dermis. In comparison, EBA has much less dermal inflammatory infiltrate [4, 7, 16].

The immunopathology findings in BSLE include direct IF with linear IgG deposits at the basement membrane and occasional granular IgA, IgM, and C3 deposits [1, 2], unlike DH and linear IgA bullous dermatosis where IgA is the unique immunoglobulin present at the direct IF. Salt-split skin indirect IF reveals IgG deposition at the dermal side of the cleavage, which is a highly suggestive finding of BSLE. In contrast, antibodies in BP are localized in the epidermal site and within the lamina lucida [4, 17, 18].

The correlation between BSLE and SLE activity remains controversial. Nonetheless, concomitant lupus nephritis, hematologic, and neuropsychiatric involvement have been reported [1, 3, 5]. In a multicenter retrospective study on BSLE patients by de Risi-Pugliese et al., lupus nephritis was reported in 50% of the cases, mostly class III or IV, according to the International Society of Nephrology/Renal Pathology Society (ISN/RPS) classification. Hematological involvement was found in 45% of cases at the time of BSLE. Moreover, the median systemic lupus erythematosus disease activity index 2000 (SLEDAI-2K) score at the time of BSLE diagnosis was 9 (3–23), suggesting the presence of moderate to severe SLE disease in these patients [5]. Interestingly, our patient presented a SLEDAI-2K of 22 and developed both severe renal compromise with nephrotic syndrome and urine sediment highly suggestive of lupus nephritis treated with mycophenolate mofetil and warm autoimmune hemolytic anemia requiring rituximab. All of these support that BSLE could be a marker of disease activity. It is believed that the neutrophils infiltrate seen in BSLE could play an important role in the severity of the disease and extra-articular manifestations, as it has been described in patients with lupus nephritis [19].

Dapsone is considered the treatment of choice for BSLE, due to its anti-inflammatory properties and the rapid improvement of the skin lesions once initiated. Doses can vary from 25 to 200 mg daily. The reappearance of new lesions has been reported in some cases where dapsone was discontinued [1, 3, 8]. However, there is evidence supporting that dapsone can be stopped after one year of treatment with no recurrence of new skin lesions [1]. The use of steroids and other immunosuppressants such as methotrexate, azathioprine, cyclophosphamide, mycophenolate mofetil, and intravenous immunoglobulin is indicated in combination with dapsone when systemic symptoms of SLE are present [3, 6–8, 20, 21]. In cases of dapsone contraindications, the literature suggests the use of steroids and immunosuppressant agents only [1, 5]. There are two different dapsone contraindications: absolute and relative. The absolute contraindications include agranulocytosis and hypersensitivity syndrome, whereas the relative contraindications include allergy to sulfonamides antibiotics, significant cardiopulmonary disease, significant liver and renal impairment, or pre-existing peripheral neuropathy [22]. The most frequent side effects of dapsone reported in patients treated for BSLE include hemolytic anemia, hepatitis, and hypersensitivity syndrome [5].

There is a concern for dapsone and sulfa cross reactivity. However, most of the studies evaluating the cross reactivity between these two drugs have been performed in HIV patients only and have shown mixed results [23, 24]. In a retrospective study evaluating the safety of dapsone in HIV patients, May S. et al. determined that dapsone is safe to use in patients intolerant to trimethoprim-sulfamethoxazole; however, 10% of patients were not included in the analysis as they had already reported dapsone intolerance [24]. In our case, the limited evidence on sulfa cross reactivity with dapsone, the relative contraindication due to this patient allergy reaction, and the need for prolonged therapy prompted us to perform desensitization before the initiation of treatment, essential for the recovery of the patient. To our knowledge, this is the first case report supporting the use of dapsone for the treatment of BSLE in patients with sulfa allergy.

The worsening of the anemia after the initiation of dapsone therapy was concerning for hemolysis related to dapsone, but further workup revealed a positive Coombs test suggestive of warm autoimmune hemolytic anemia in the setting of active SLE, successfully treated with rituximab, which has shown to be of benefit in refractory BSLE cases as well [8]. In our patient, rituximab was added one week after dapsone for warm autoimmune hemolytic anemia with further improvement of anemia and skin lesions. Therefore, it is unclear if rituximab (375 mg/m^2^ protocol over 4 weeks) added an extra benefit to dapsone in this particular case.

## 4. Conclusions

This case highlights the importance of the prompt recognition of BSLE as the initial manifestations of SLE and its association with high disease activity with a concomitant risk of lupus nephritis and autoimmune hemolytic anemia. This is the first case reported in the literature that describes a patient with BSLE and sulfa allergy who presented with refractory disease to multiple immunosuppressant agents and rapid resolution of blistering lesions after the initiation of dapsone. Therefore, this case emphasizes the use of dapsone as the cornerstone of treatment of BSLE, even in the presence of sulfa allergy and systemic SLE symptoms.
